# Emerging role and therapeutic application of exosome in hepatitis virus infection and associated diseases

**DOI:** 10.1007/s00535-021-01765-4

**Published:** 2021-03-04

**Authors:** Ying Shi, Lingyao Du, Duoduo Lv, Yan Li, Zilong Zhang, Xiaolun Huang, Hong Tang

**Affiliations:** 1 Center of Infectious Diseases, West China Hospital of Sichuan University, No. 37 Guoxue Alley, Wuhou District, Chengdu, 610041 Sichuan China; 2 Division of Infectious Diseases, State Key Laboratory of Biotherapy and Center of Infectious Diseases, West China Hospital, Sichuan University, No. 17 People’s South Road, Chengdu, 610041 Sichuan China; 3 School of Medicine, University of Electronic Science and Technology of China, No. 4 Section 2, North Jianshe Road, Chengdu, 610054 Sichuan China; 4 Department of Hepatobiliary Surgery and Cell Transplantation Center, Sichuan Academy of Medical Sciences and Sichuan Provincial People’s Hospital, No. 32 Western Section 2, 1st Ring Rd., Chengdu, 610072 Sichuan China

**Keywords:** Exosome, Hepatitis virus, Immune escape, Immune response, Exosomal nanoshuttle

## Abstract

Hepatitis viruses are chief pathogens of hepatitis and end-stage liver diseases. Their replication and related pathogenic process highly rely on the host micro-environment and multiple cellular elements, including exosomes. Representing with a sort of cell-derived vesicle structure, exosomes were considered to be dispensable cellular components, even wastes. Along with advancing investigation, a specific profile of exosome in driving hepatitis viruses’ infection and hepatic disease progression is revealed. Exosomes greatly affect the pathogenesis of hepatitis viruses by mediating their replication and modulating the host immune responses. The characteristics of host exosomes are markedly changed after infection with hepatitis viruses. Exosomes released from hepatitis virus-infected cells can carry viral nucleic or protein components, thereby acting as an effective subterfuge for hepatitis viruses by participating in viral transportation and immune escape. On the contrary, immune cell-derived exosomes contribute toward the innate antiviral immune defense and virus eradication. There is growing evidence supporting the application of exosomal biomarkers for predicting disease progress or therapeutic outcome, while exosomal nanoshuttles are regarded as promising therapeutic options based on their delivery properties and immune compatibility. In this review, we summarize the biogenesis and secretion mechanism of exosomes, review the recent findings pertaining to the role of exosomes in the interplay between hepatitis viruses and innate immune responses, and conclude their potential in further therapeutic application.

## Introduction

Hepatitis virus infection remains a severe public health problem with considerable morbidity and mortality, leading to about 1.5 million deaths on a global scale annually [1]. Commonly, hepatitis viruses consist of five major groups: hepatitis A virus (HAV), HBV, HCV, HDV and HEV, though other hepatotropic viruses are now under continuously investigating. Prevalence of each kind of hepatitis virus shows distinct geographical localization, while HBV and HCV cause the most serious socioeconomic burdens, especially in developing areas such as those in Africa and Asia [2]. With significant advances in antiviral agents, eradication of HCV is achieved through directly acting antivirals (DAAs). However, therapy of HBV remains an unmet issue due to the limited antiviral efficacy of current anti-HBV options when facing covalently closed circular DNA (cccDNA) [3].

Hepatitis viruses utilize the host materials for replication and establish long-term localization. Their life cycle begins with attaching and entering into hepatocytes, which requires unique receptors located on the cell surface, such as sodium taurocholate cotransporting polypeptide (NTCP) for HBV and CD81 for HCV [4, 5]. Subsequently, hepatitis viruses hijack the host transcriptional machinery and cellular materials for their replication. Uncontrolled viral replication leads to massive hepatocyte necrosis and inflammatory infiltration as well as the development of severe progressive syndromes like cirrhosis, hepatocellular carcinoma (HCC), or other critical illnesses [6].

Over the past decades, extracellular vehicles (EVs) have been shown to play irreplaceable roles in the interplay between hepatitis viruses and the host immune system [7]. Derived from the fusion of multivesicular bodies (MVBs) with the plasma membrane, EVs can carry and transport biologically active molecules to target cells, deliver specific signals to regulate a wide range of processes. Development in the technology of exosome collection and purification supports to find that hepatitis viral genome or proteins can be packaged into exosomes. In pathological conditions, these specific exosomes provide a stretch for viral replication and immune escape. In this review, we summarized recent studies on exosomes related to the hepatitis viruses and reviewed the dual role of exosomes in the mutually dependent relationship between viral pathogenesis and immune response.

## The essence of exosome

EVs represent a broad category of cell-derived particles coated with a lipid bilayer, which can be classified into exosomes, microvesicles, and apoptotic bodies based on their modes of biogenesis. Exosomes are endosomal-origin phospholipid nanovesicles with a typical cup-shape morphology and are 30–150 nm in diameter, which can be found in almost all eukaryotic fluids, like blood, urine, and cultured medium of cell cultures, and can be isolated using their specific sedimentation properties with a high-velocity centrifuge or an organic solvent-aided sedimentation process [8, 9]. The composition of exosomes derived from various cell sources varies in quantity and type, while several conserved protein groups have been identified as universal markers based on mass spectrometry-based proteomic and lipidomic analyses. An example is the tetraspanin family of proteins, also known as the transmembrane 4 superfamily (including CD9, CD63, and CD81 among other members) comprising four hydrophobic domains, which are embedded in the membrane of various cells. Their conserved transmembrane structure forms channels to connect the inside of the cells with the outside environment [10]. Heat shock proteins (HSP70, HSP90) are also ranked among the top exosomal markers, which assist membrane remodeling by regulating protein folding and transformation. Other common exosomal components like MVB biogenesis molecules (Alix and TSG101) [11] and GTPases exist in universal structural components of almost all exosomes, and are considered to be common exosomal markers [12]. Specific cell-derived exosomes contain distinct molecules, for example, MHC class I and II are more commonly seen in exosomes released from B lymphocytes, T lymphocytes, and dendritic cells (DCs) [13]. Except for surface protein markers, the lipids or lipoid components are essential components involved in membrane trafficking, antigen presentation, target adhesion, and so on. The classical components of the exosomal membrane include cholesterols, ceramides, lipid rafts, and sphingomyelin that form the basic bilayer membrane structure [14]. Besides, the administration of palmitate or its metabolite, lysophosphatidylcholine, has also been suggested to contribute in the release of exosomes [15].

**Fig. 1:**
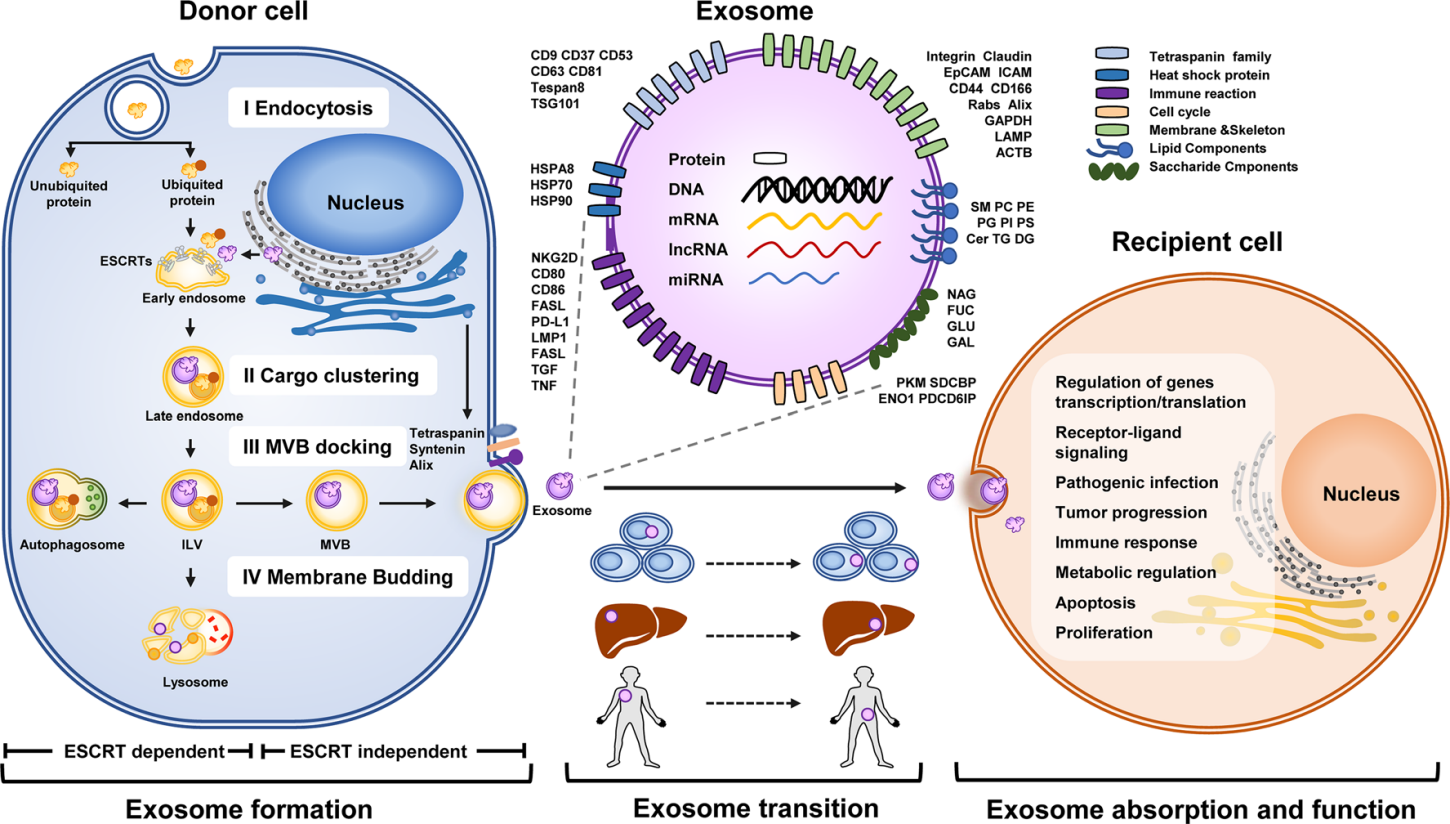
Biogenesis and function of exosomes. During ESCRT-dependent exosome formation: I. proteins are endocytosed from plasma membrane to be incorporated into endocytic vesicles. Ubiquited proteins combine with ubiquitin-binding domain of ESCRTs on early endosome; II. Proteins are sorted via late endosome and intraluminal vesicle; III. A portion of these cargos transport MVBs to dock on the plasma membrane, while the rest are fused with autophagosome and lysosome to degradation; IV. MVBs fuse with the plasma membrane and bud their contents out. Meanwhile, distinct transmembrane proteins are recruited to the plasma membrane where MVBs are docking, helping the final formation and release of exosome in an ESCRT-independent way. Exosome released from donor cells can be absorbed directly by the adjacent recipient cells or undergoes the paracrine pathway and endocrine pathway. After being absorbed into recipient cells, exosome bud contents out to participated in various biological processes

Exosomes originate from membrane cavities or early intracellular bodies, which sag inward to form the endovascular vacuoles and MVBs, following which the MVBs fuse with the cell surface under the traction of intracellular molecular motors to release the exosomes. Some components sorted by the endosome can be presented in this process for exosome packaging. Biogenesis of exosome involves multiple membrane rupture or formation processes and is under the control of the cellular endosomal sorting complex required for transport (ESCRT) pathway through the regulation of the sorting ubiquitinated endocytic cargo (Fig. 1) [16, 17]. ESCRT machinery is composed of four heterodimeric subcomplexes, including ESCRT-0, ESCRT-I, ESCRT-II, and ESCRT-III; each complex has a unique accessory component of vacuolar protein sorting-associated protein (VPS). ESCRT machinery are employed in the inward budding of endosomal membranes; ESCRT-0, ESCRT-I, and ESCRT-II complexes act consequently to sort the ubiquitinated cargo via their ubiquitin-binding domains, and distinct transmembrane proteins are recruited to activate ESCRT-III, which are then incorporated into the invaginated membrane. The cytosolic components are engulfed within the ILVs (intraluminal vesicles), this process is affected by lysobisphosphatidic acid (LBPA) following the correct incorporation of MVBs [18–20]. Part of intracellular MVBs are transmitted to the lysosomes where they are subjected to proteasomal degradation, following which they fuse with lysosomes [21]. Alternatively, a portion of MVBs fuse with the plasma membrane and bud their contents out, which are defined as exosomes [22, 23].

Once released into the external environment, exosomes can be absorbed directly by the adjacent recipient cells, by cells far apart through the paracrine pathway, or they can be circulated before being absorbed through the endocrine pathway. The host-derived element on the surface of the exosome protects it from immune elimination and supports its separation in almost all eukaryotic fluids, such as blood, urine, and also the cultured medium of cell cultures in vitro. When accessing the recipient cells, most exosomes are recognized by cell adhesion factors such as integrin followed by endocytosis, while some can directly fuse with the recipient cell membrane. In certain cases, the transmembrane protein on the exosome can target factors located on the surface of the recipient cell, thereby initiating the signal transformation process.

Exosomes that were initially regarded as a cellular waste have now been shown to play key roles in various biological processes. Exosomes dispose waste products to maintain cellular homeostasis in donor cells and regulate numerous physiological and pathological processes in recipient cells, thereby exerting a bilateral function. The role of exosome is more complicated in morbid status as they express only selective contents compared to that in the normal condition, especially in tumor status and pathogenic infection. Numerous viruses, such as paramyxoviruses, rhabdoviruses, herpesviruses, filoviruses, and arenaviruses, can exploit the ESCRT machinery to mediate the formation and release of infectious particles directly [24]. Nevertheless, exosomes may act as antigen-presenting vesicles to participate in the immune response, and affect the progression of multiple virus-related diseases. Several studies have demonstrated that exosomal miRNAs derived from hepatitis virus-infected cells can regulate the expression of target genes in the initial stages of liver fibrosis and carcinogenesis [25].

## Exosomes in hepatitis virus infection

### Exosomes participate in life cycle of hepatitis viruses

Accelerating studies have unearthed that exosome can serve as viral carriers, thereby contributing to viral replication or pathogenesis directly. The first evidence comes from the understanding that components of ESCRT are required during viral capsid packaging and the maturation of numerous enveloped viruses [26, 27]. In addition to the classical replication pattern, current research illustrated the viral component to exist in the exosome, thereby demonstrating that hepatitis viruses adopt the exosomes to transmit their genome and establish productive infection (Table 1). For those viruses, it is a viable way to hide within exosomes and escape from immune detection through the ESCRT-dependent viral budding mode [28].

**Table 1:** Function of hepatitis viruses induced exosome

Viral type	Containing factor	Donor cell	Recipient cell	Biological function	References
HBV	RNA	Hepatocyte	NK cells	Promote innate anti-HBV immune response	[70]
	DNA	Hepatocyte	Hepatocyte	Resistant to antibody neutralization	[40]
	DNA	Sera of CHB patients	Hepatocyte	NK-cell dysfunction	[39]
	RNA	Hepatocyte	THP1 cell	Activate innate immunity to restrict HBV replication	[114]
	HBx protein	Hepatoma cell	Hepatic stellate cell	Influence hepatic microenvironment	[41]
	DNA	CHB patients	Naïve hepatocyte cell lines	Transmit HBV to primary hepatocytes	[37]
HCV	RNA	HCV-infected hepatocytes	Monocyte	Promote macrophage polarization and circulating fibrocyte generation	[86]
	RNA	Hepatocyte	pDCs	Activate pDCs and promote HCV infection	[82]
	RNA	Sera of HCV- infected patients/hepatocytes	Hepatocytes	Transmit HCV transfection	[115]
	RNA	Hepatocyte	pDC	Activate innate immune and type I IFN production	[116]
	RNA	Hepatocyte	DC	DCs mature to induce NK and CTL activation	[80]
	RNA	Hepatocyte	pDC	Trigger innate immunity	[82]
	RNA	Hepatocyte	Monocytic myeloid cell	Trigger the expansion of myeloid-derived suppressor cells	[117]
	dsRNA	Hepatocyte	Hepatocyte	Reduce the activation of toll-like receptor 3	[97]
	RNA	Hepatoma cell	Hepatoma cell	Transmit productive HCV infection and are partially resistant to antibody neutralization	[45]
HAV	eHAV	Hepatocyte	pDC	Produce substantial IFN-α to active innate immune	[79]
HEV	RNA	Hepatoma cell	–	Escape from the immune response	[118]
	viral ORF3 protein	Hepatoma cell	Hepatoma cell	Modulate the host response	[58]

Exosome containing viral components (protein, nucleic acids) are categorized with their types, donor cells, recipient cells, and biological function as listed

#### Exosome mediates HBV replication and transition

HBV infection results in about 257 million chronic hepatitis patients and 887,000 annual deaths worldwide [29]. The virion is a para-retrovirus containing a partial double-stranded and relaxed circular DNA (rcDNA) [30]. Once being engulfed by hepatocytes, rcDNA get fixed and conversed to cccDNA and transcribed into HBV RNAs with different lengths and roles, such as the pregenomic RNA (pgRNA) and precore mRNA [31]. pgRNA encodes core protein and P protein to assemble the core particle of HBV, while precore mRNA encodes the secretory protein HBeAg. Subsequently, HBV core particle is completely packaged into an icosahedral capsid to form a replication intermediate sphere with a diameter of 42 nm, then being released out from the original infected cells [32]. Persistence of cccDNA results in long-term chronic infection and treatment failure with nucleos(t)ide analogues (NA) and pegylated-interferon (PEG-IFN) in chronic hepatitis B (CHB) patients.

During the formation of HBV virions, multiple host proteins are expropriated by HBV to catalyze membrane fission, virion package, or release sorting signals. With the high comparability in shape and particle structure, the steps of HBV budding and exit from host cells are associated with ESCRT. Previous studies have confirmed that changes in the microenvironment of HBV infected hepatocytes include factors involved in exosome morphogenesis and protein secretion, such as Annexin A1/A4, COPB1/2, and vesicle-associated membrane protein-associated protein A (VAPA) [33]. Both the whole viral particle and single viral proteins can trigger exosomal protein expression. For example, taxilin alpha (TXLNA) -mediated interaction between HBsAg and the ESCRT component, TSG101, promotes the secretion of viral sub-particles containing liver-specific miRNAs [34, 35]. Likewise, the HBx protein can interact with exosomal biomarkers, such as CD9 and CD81, by utilizing the ESCRT machinery to enhance exosome secretion. Furthermore, the extracellular matrix of HBV infected cells is greatly reshaped compared to normal cells. Quantitative proteomic analysis revealed that HBV or HBx not only changed the exosome contents but also influenced key factors of the extracellular matrix, such as vimentin (VIM) and keratin (KRT)18, inducing re-construction of local hepatic structure and fibrosis directly [36].

Exosome acts as a powerful carrier of viral DNA and protein components, shuttling them from infected to non-infected cells to facilitate HBV spread. HBV virions have been observed to be localized to the membranes of the late endosomes and large intracellular compartments, indicating ESCRT effect in ensheathing HBV somehow [37]. Deficiency of ESCRT impairs HBV budding and/or release. The first evidence comes that when essential genes of ESCRT like actin-interaction protein (AIP) 1 or VPS4B were mutated, production of MVB was attenuated and inhibited extracellular enveloped HBV virions [38]. A more direct evidence is that HBV components was detected in exosomes purified from the serum of CHB patients, including nucleic acids like cccDNA and rcDNA as well as viral proteins like HBx and HBsAg [39]. Abundant HBV DNA has been detected in exosomes isolated from HBV-infected primary hepatocytes [40]. Moreover, both HBx mRNA and protein were found to be packaged in exosomal cargos, protecting them from the host nuclease attack [41].

#### Exosome mediates HCV replication and transition

HCV is another major type of hepatitis virus infecting170 million people worldwide. The genome of HCV, a positive single-stranded RNA, encodes essential components for viral particle formation including three structural proteins (core, E1, and E1) and seven nonstructural proteins (p7, NS2, NS3, NS4A, NS4B, NS5A, and NS5B) after entry into the hepatocytes [42, 43]. The positive-strand RNA serves as a template to synthesize the replication intermediates as well, particularly the double-stranded RNA (dsRNA); then, the synthetic negative-strand RNA replicates to synthesize a bunch of progeny genomic RNA. With the help of cellular apolipoproteins, a replicase complex is assembled within vesicle-like membranes, and ultimately formatted into cell-accessible viral particles [44].

Similarly, exosomes derived from HCV-infected hepatocytes are observed to contain complete or subgenomic HCV RNA, validated in both laboratory models and clinical samples from HCV-infected patients [45, 46]. Depletion of ESCRT component led to a significant reduction in exosome production, sequentially resulting in decreased HCV replication [47]. The replication-competent genomic RNAs in exosomes are crucial in HCV secretion and cell to cell transmission [48]. Interestingly, the secretion of HCV-containing exosomes requires a tetraspanin, CD81, which facilitating the viral attachment and fusion by mediating the recognition to HCV envelope glycoprotein E2 [49, 50]. In the absence of CD81, HCV envelope proteins are almost completely retained in the endoplasmic reticulum [51]. Correspondingly, treatment with U18666A, an MVB transport inhibitor, led to an increased viral particle accumulation in cells but less secretion, indicating that the endosomal pathway is involved in HCV particle release [52].

#### Exosome mediates HAV/HEV replication and transition

HAV and HEV have also been reported to egress virus particles in an exosome-mediated way [53, 54]. HAV is a type of picornavirus lacking envelope; capsid formation of HAV relies on ESCRT III-dependent process [55]. HAV released from cells is cloaked in host-derived membranes, which facilitates their escape from neutralizing antibodies and promotes virus spread [56]. The phosphatidylserine receptor, hepatitis A virus cellular receptor 1 (HAVCR1), and the cholesterol transporter, Niemann-Pick disease type C1 (NPC1) participate in cargo delivery of exosomes from HAV-infected cells which contains HAV RNA to cytoplasm by endocytosis [57].

Similarly, HEV existing in the blood is membrane-associated and in a quasi-enveloped form compared with the naked wildtype HEV. As particles released from infected cells via the exosomal pathway, the lipid membrane of HEV capsids resembles the membrane of exosomes [58]. Rab5 and Rab7, two proteins regulating exosome production and secretion, are required for enveloped HEV transportation while blocking endosomal acidification abrogates HEV production and infectivity [59].

### Exosomes participate in hepatitis virus-related liver diseases

Uncontrolled viruses accumulate risk of fibrosis, cirrhosis and carcinogenic process consequently. Exosome-mediated viral-transportation helps to circumvent the supervision of immune system and stimulate morbigenous pathways. In addition, an exosome is required in the transition of multiple phlogogenic or oncogenic factors. In hepatitis virus-induced inflammatory state, damaged parenchymal hepatic cells secrete inflammatory factors, such as platelet-derived growth factor (PDGF) and transforming growth factor-β (TGFβ), resulting in the activation of hepatic stellate cells (HSCs) from quiescent condition to initiate the fibrogenic stage. The most significant symbol of hepatic fibrosis is intrahepatic connective tissue dysplasia and a massive diffuse extracellular matrix (ECM) deposition, which is labeled with the upregulation of collagen, laminin, α-smooth muscle actin (α-SMA), and so on. Growing evidences have clarified that exosomes from hepatitis virus-infected hepatocytes regulate their contact with HSCs [60]. Exosomes derived from HCV-infected hepatocytes contain miR-19, which are then directly internalized to modulate SOCS-STAT3 axis and upregulate ECM factors in HSC [61]. TGF-β is recognized as an effector involved in the activation of fibrosis through increasing SMAD-dependent transcription. TGF-β, which is hardly expressed in normal conditions, is found to be increased when exposed to hepatitis viruses or even viral elements [62]. miR-192 is also found in exosomes from HCV-infected hepatocytes, which is transferred to HSCs to upregulate TGF-β1, resulting in the activation and transdifferentiating of HSCs into myofibroblasts [63]. TGF-β2 released from HCV-infected cells is passed on to HSCs in an exosome-mediated autocrine manner, leading to an increase in fibrogenic responses in the adjacent HSCs [64].

Persistent active viral replication is responsible for irreversible tumorigenic progression, indicating HBV- or HCV-related HCC. During HBV infection, factors accounting for hepatocarcinogenesis include the integration of HBV DNA, the oncoprotein HBx and preS/S, and HBV-inflicted DNA damage due to hepatocellular regeneration [65]. Exosomes carrying these oncogenic elements may activate some carcinogenic pathways like the classical phosphatidylinositol 3-kinase (PI3K) or mitogen-activated protein kinase (MAPK) pathways to trigger the carcinogenesis process in target cells. Moreover, HCC-derived exosomes possess distinct contents, which account for tumor malignancy, metastasis, immune escape, and drug resistance. Epithelial-mesenchymal transition (EMT) process is positively correlated with the degree of tumor metastasis to a significant extent; tumor cells undergoing EMT can release exosomes containing components beneficial for tumor transition. miRNA is a type of endogenous non-coding RNA with a small size (19–23 nts), which competitively modulates its target gene expression by silencing transcription. A set of miRNAs targeting multiple oncogenes or tumor suppressor genes (hsa-miR-125b-5p, hsa-miR-374a-5p, hsa-miR-24-3p, hsa-miR-200b-3p, and hsa-miR-21-5p) are statistically upregulated in the exosomes from EMT-hepatic cells [66].

## Exosomes in the interplay between hepatitis viruses and the immune system

**Fig. 2:**
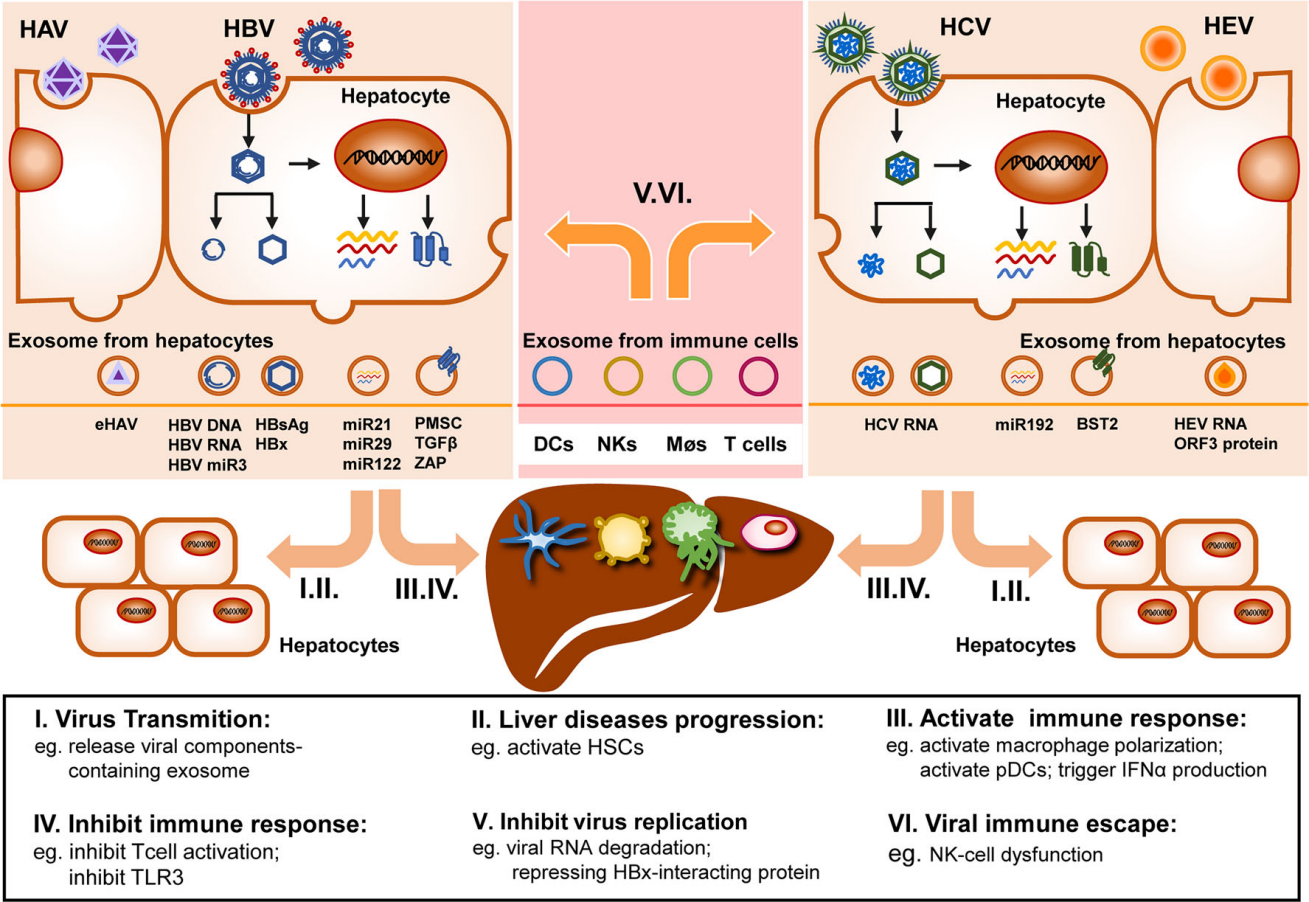
Exosome-mediated immune evasion of hepatitis viruses. Different origins and contents endow exosome with variable function: exosomes from infected hepatocytes can transmit infective viral component, hiding them from the immune system (I, IV); exosome containing specific product from infected hepatocytes can induce immune response and liver diseases progression in target cells (II, III); almost exosome released from immune cells transmits antiviral factors, while immune-inhibitory factors can also be found inside (V, VI)

The complicated microenvironment endows exosomes with more paradoxical characters to the virus in liver: friend and foe. Apart from broadening the transmission of hepatitis viruses, particles carrying increased viral antigens also facilitate their immunogenicity and elicit amplified immune reaction concomitantly [67]. Normally, hepatitis viruses can be recognized by various immune cells through the pattern recognition receptors (PRRs), ensuing the activation of the innate immune response. Effective innate immune responses to viral invasion are necessary in viral pathogenesis or clearance, which mainly occur in natural killer (NK) cells, dendritic cells (DCs), and T cells via the activation of interferon-related signaling pathways (Fig. 2) [68]. But in some conditions, exosomes can also contribute to immune escape and make the immune interaction more elusive, especially in this immune privilege organ: “liver”.

### Immune cell-derived exosomes regulate the host response to hepatitis virus invasion

NK cell is a type of cytotoxic lymphocyte implementing immunological surveillance in the non-specific immune response of the host via its cytotoxic function or secretion of specific antiviral immune cytokines. The biofunction of NK cell is complex. An essential immune-activating receptor, natural killer group 2 member D (NKG2D), in NK cell regulates its antiviral process predominantly. NKG2D combines with its ligands to distinguish abnormal cells and transduce the anti-viral signal. Proteins carried on the exosome from morbid cells, such as HSP70, can directly activate the cytolytic and migratory capacity of NK cells [69]. Exosomes released from HBV-infected hepatocytes contain HBV nucleic acids, which can stimulate MyD88, Toll-IL-1 receptor-containing adaptor molecule-1 (TICAM-1), and mitochondrial antiviral signaling (MAVS) -dependent pathways to induce NKG2D ligand expression, and evoke NK cells [70]. Both resting and activated NK-cells can release exosomes displaying immune homeostatic activities [71]. A sets of typical NK cell markers and antiviral proteins have been found to be expressed on NK cell-derived exosomes, such as perforin and natural cytotoxicity receptors (NCRs, i.e., NKp30, NKp46, and NKp44) [72, 73].

DCs are the most effective antigen-presenting cells when encountering viral attack, and stimulate the initial T cell activation or NK function through the Toll/IL-1R domain-containing adaptor-inducing IFN-β (TRIF) pathway [74, 75]. Recognition of hepatitis virus is the first step to induce DC maturation and produce endogenous type I interferon that initiates and triggers the innate immune responses [76, 77]. HBV DNA and HBsAg can be recognized by TLR9 on DCs, and then be processed by functional T cells [78]. HCV or HAV are sensed by plasmacytoid DCs (pDCs) for peak immune response [79]. Exosomes containing HCV RNA purified from the supernatant of HCV-infected hepatocytes led to TLR3-mediated DC maturation [80]. Furthermore, DC-derived exosomes, named as DEX, which contain the surface expression of functional major histocompatibility complex (MHC)–peptide complexes, are under trial for the feasibility of DC-based immune therapy [81]. Previous research has explored that when countering viral infection, genes related to exosome trafficking, such as *Charged Multivesicular Body Protein 4B (CHMP4B), TSG101, and Annexin A2 (ANXA2)* are activated in pDCs [82]. DEX harboring IL15Rα is considered to boost NK and T cell immune responses via IFNγ secretion [83]. In phase II clinical trial, IFN-γ-containing DEX was observed to upregulate NKp30 ligand B cell lymphoma 2 (Bcl2)-associated athanogene cochaperone 6 (BAG6) and MHC class II level in patients bearing inoperable non-small cell lung cancer without tumor progression, who achieved longer progression-free survival compared to the control groups [84].

Macrophage is a type of white blood cell that can digest cellular debris or other foreign substances and produce inflammatory cytokines. The liver-resident macrophages are defined as Kupffer cells [85]. During hepatitis virus infection, TLRs are crucial in mediating monocyte differentiation and macrophage polarization [86]. Macrophage-derived exosomes can access the hepatocytes through T cell immunoglobulin and mucin receptor 1 (TIM-1), and then deliver IFN-α-induced anti-HBV activity [87]. In HCV infection, the number of activated macrophages increased as well as the TNF secretion. Activated macrophages also conferred anti-HCV activity to hepatocytes via the consequent release of exosomes containing anti-HCV miRNA-29 family members [88, 89].

### Hepatitis virus achieves immune escape via exosomes

Apart from using the ESCRT machinery to assemble and transmit virions, these tiny but proficient viruses also rely on exosomes to puzzle and escape the host immune defense. Normally, these pathogens could stimulate the maturation of DCs, T cells, and other immune effective cells, which correspondingly secrete inflammatory factors to inhibit different stages of viral replication or directly eliminate the infected hepatocytes [90]. However, when endogenous immune system was insufficient for viral clearance, it would lead to chronic infection and the risk of end-stage liver diseases in some cases. Although the viral exosomes enhance the visibility of the viruses to the innate interferon pathway, recent studies have demonstrated that the hepatitis virus can escape the host immune defense by impairing the DC function [91]. In patients with chronic HBV infection, both the quantity and function of pre-DCs or mature DCs are impaired [92, 93].

Stimulating a set of immune inhibitory factors is another approach for hepatitis viruses to achieve immune escape. Previous research has shown that HBV infection could upregulate representative immunosuppressive factors, such as TGF-β1, thereby blocking NKG2D and 2B4 activity resulting in NK-cell dysfunction [94]. Consistent with this finding, NK cells were also functionally impaired by persistent HBV-positive exosomes with decreased cell cytotoxicity and IFN-γ production [39]. Exosomes produced by HBV-infected cells were found to suppress the retinoic acid-inducible gene I (RIG-I) on NK cells, which dampened the nuclear factor κB (NF-κB) and p38 mitogen-activated protein kinase pathways. An ISG-coding protein, IFITM2, was found on exosomes secreted by HBV-infected hepatocytes; they were enrolled in the negative feedback regulation of the IFN pathway by attenuating the IRF3/TBK1-mediated IFN pathway after targeting DCs [95]. Besides, HBV facilitates the externalization of some antiviral proteins, like APOBEC3G, by assembling them into exosomes, followed by the corresponding decrease in the intracellular level of the restriction proteins [96].

Similar immune escape mechanisms occur in HCV-infected cells. HCV-infected cells substantially secrete exosomes containing HCV RNA. These exosomes target the surrounding cells to reduce the activation of toll-like receptor 3 (TLR3) and interfere with the anti-viral ISG activation. Blocking exosome secretion by GW4869 reanimated the TLR3-related pathway and elicited subsequent antiviral response [97]. These HCV RNA-containing exosomes were found to be associated with T cell activation by inhibiting IL-2 release and CD69 expression after co-culturing [98].

## Exosomes serve as promising therapeutic strategy

### Exosomal biomarkers in viral hepatitis and related liver diseases

There is a tight link between hepatitis infection with the hepatic histopathological change, even tumorigenesis. However, current imaging techniques, including ultrasonography and magnetic resonance are still insufficient for precise diagnosis in patients with asymptomatic early-stage HCC, or prediction of therapeutic outcomes. Therefore, biomarkers with both accuracy and accessibility are urgently needed. Now that heterologous exosomes can carry proteins or nucleic acid species of originated cells, identifying distinct disease-related biomarkers might contribute towards the identification of risk patients for the outbreak even before the initial symptoms [99]. Updating researches on EVs and their specific signatures provide a theoretical basis and guide future clinical investigation (Table 2).

**Table2:** Hepatitis viruses-related exosome involved in liver environment

Biomarker	Donor cell	Recipient cell	Function	References
PMSC1/2	Hepatoma cell	Monocyte	Induce production of pro-inflammatory molecules	[101]
PMSD1/7/14				
DDX60	Hepatocyte	NK cells	Mediated cytoplasmic viral HBV RNA degradation	[70]
miR-192/92a/200b	Plasma	HSC	Down-regulated during HSC activation	[119]
TGF-β	Hepatoma cell	Hepatoma cell	Induce TGF-β mediated suppression of HBV	[120]
HBV-miR-3	Hepatocyte	Hepatocyte	Attenuate HBV replication and Repress HBsAg/HBeAg	[121]
miR-122	Hepatoma cell	Hepatoma cell	Inhibit HBV expression	[122]
miR-21/ 29	Hepatocyte	NK cells	Inhibit NK cells and suppress HBV proliferation	[104, 123]
ZAP	Hepatocyte	Hepatocyte	Degrade HBV pgRNA substrate and control HBV replication	[124]
OSTM1	Hepatoma cell	Hepatoma cell	Downregulate HBV replication through posttranscriptional regulation or RNA stability	[125]
miRNA204	Hepatoma cell	Hepatoma cell	Suppressive effect on HBV replication	[126]
Ski2	Hepatocyte	Hepatocyte	Negatively regulate HBx mRNA, suppress HBV replication	[127]
miR-15b	Hepatocyte	Hepatocyte	Promote HBV replication by aiding HBV enhancer I activity HNF-1α	[128]
APOE	Hepatocyte	Hepatocyte	Promote hepatitis B virus infection and production	[129]
APOBEC3G	Hepatocyte	Hepatocyte	Inhibits HBV replication	[130]
IFITM2	Hepatocyte	Dendritic cell	Inhibit IFNα pathway activation and block anti-HBV efficacy of exogenous IFNα	[95]
CD81+	Hepatocyte	Macrophage	Carry HCV particles and establish persistent infection	[131]
Ago2-miR122-HSP90	Serum or Hepatoma cell	Hepatoma cell	Enhance HCV RNA stability and viral replication	[115]
miR122	Hepatoma cell	Hepatoma cell	Targets CCNG1 and NDRG3 to inhibit viral replication	[132]
IFITM1	Hepatocyte	Hepatocyte	Interruption of viral coreceptor function	[133]
GAL-9	Monocytes	T cell	Inhibit T cells in HCV infection	[134]
ISG	LSEC	Hepatocyte	Inhibit HCV replication	[135]
miR-192	HCV-replicating hepatocyte	HSC	Activation and transdifferentiation of HSCs into myofibroblasts	[63]
miR-19a	Hepatocyte	Hepatic stellate cell	Activate HSC by modulating the SOCS-STAT3 axis	[61]
UCHL1	HCV-infected hepatocyte	HSC	Stimulate HSCs activation through JNK phosphorylation	[136]
miR-501	Hepatoma cell	Hepatoma cell	Activate HBV replication by repressing HBx-interacting protein (HBXIP)	[137]
miR-125b	Hepatoma cell	Hepatoma cell	Inhibit HBV DNA intermediates and the secretion of HBsAg and HBeAg by targeted repression of SCNN1Α	[138]
BST2	Hepatoma cell	Hepatocyte	Inhibit HCV assembly or release	[139]
miR-29	Macrophage	Hepatocyte	Activate macrophage and inhibit HCV replication	[89]

Exosome secreted from different types of cell under hepatitis virus infection are summarized, which are labelled with corresponding donor cell, recipient cell and biological function

In recent years, a set of microRNAs (miRNAs) are found to be greatly involved in hepatitis virus replication and transition. Hepatitis virus infection affects the miRNA levels and proteins in EVs released from virus-infected cells that transport from tissue to serum to regulate the host innate immune system [100, 101]. Expression of miRNA clusters significantly differed in the tissues of HBV- and HCV-infected individuals, as well as in the exosomes [102]. For example, either HBV infection or HBX overexpression could induce miR-21 and miR-29 expression in HCC cell lines, followed by a simultaneous increase of exosomal miR-21 and miR-29 levels [70, 103]. miR-21 and miR-29 directly target IL-12 subunits to reduce IL-12, a heterodimeric cytokine secreted by DCs or macrophages to activate NK cells, indicating that HBV counteracts the host innate immune responses by inducing exosomal miR-21 and miR-29 to attenuate the IL-12 production [104]. Besides, increased exosomal miR-21 is more frequently observed in HBV-related cirrhosis and HCC patients, and may act as a promising biomarker in the diagnosis of early-stage HCC [105].

In contrast with morbigenous role in HBV infection, exosomal miR-29 exerts an anti-viral effect during HCV infection. In HCV-infected individuals, exosomes released from macrophages were found to contain miR-29 family members, while inhibiting miR-29 led to the direct restoration of HCV replication [89]. Further analytical studies showed that HCV-induced miR-122 levels varied among different HCV genotypes. In addition, miR-122 levels in the serum and exosomes were both higher in patients that achieved SVR than that in patients who did not achieve SVR, indicating that the serum and exosome miR-122 might reflect viral hepatitis therapeutic efficacy [106]. These observations suggest that distinct exosomal contents are closely associated with diverse disease conditions or viral status, and their roles differ with different pathogen infection.

### Exosome is a potential therapeutic tool in hepatitis virus-associated liver diseases

Exosome has an excellent therapeutic application in hepatitis virus infection due to its carrying capacity, self-toleration, bio-safety, resistance to RNases and proteases [107]. Engineered exosomes loaded with HBV antigens can be internalized by antigen-presenting cells, act as a decoy to induce cross-priming and antigen-specific cytotoxic T lymphocyte (CTL) immunity [108]. Uploading HCV NS3 on exosomes resulted in the successful activation of CD8^+^ T cells via DCs [109]. These findings promote the optimization of exosome-based anti-hepatitis virus agents for both curative and economic consideration, even though HCV is curable with DAAs.

Artificial exosomes can also serve as ideal vehicles for therapeutic protein, nucleic acid, and drug delivery based on the nano perforation technique. For instance, the CRISPR/Cas9 system is an accurate genomic editing approach, guided by a short gRNA sequence that attaches (binds) to a specific target DNA sequence. The functional components of gRNA and Cas9 protein have been validated to be loaded into the exosome, supporting to transfer the genetic editing effect. Naturally produced endogenous exosomes have been successfully used to deliver the functional Cas9 and HBV-specific gRNA to cut HBV DNA transfected in Huh7 cells [110].

Exosome derived from specific tumor cells (TEX) and immune cells are promising therapeutic materials due to their direct or indirect immune-regulatory effect. Exosomes secreted from umbilical mesenchymal stem cells (uMSCs) were found to inhibit HCV infection in vitro, especially viral replication, with low cell toxicity [111]. TEX-pulsed DCs (DC-TEX) induce antitumor responses and change the tumor microenvironment by decreasing regulatory T cell (Treg) accumulation in the tumor tissue [112]. DEX is another therapeutic candidate as a cell-free immune vaccine in HCC treatment. DCs enriched with AFP have been shown to elicit strong antigen-specific immune responses, retarded tumor growth, and significantly prolonged survival rates in an HCC model [113].

## Conclusion

Acknowledged with the remarkable role in cell–cell communication, exosomes have been shown to play a role in the life cycle of hepatitis viruses and affect the pathogenesis of viral hepatitis. Exosomes containing viral components directly enhance viral infection by accelerating viral transmission to uninfected cells. Hepatitis viruses cloak their full-length or fragile hereditary elements into exosome particles, thereby becoming invisible to the host immune system. These autologous membrane structures facilitate viral transmission by protecting these viruses from neutralizing antibodies and assisting their dissemination within the host, thus providing a basis for persistent infection. In this review, the dual roles of exosomes in viral hepatitis replication and immune response is summarized. Further studies are needed for clarifying the exosomal content and the biological function of exosomes in viral hepatitis, which will help better understand the pathogenesis, provide reliable predictive factors for prognosis, and aid in the development of novel therapeutic strategies.

## Abbreviations

cccDNA: Covalently closed circular DNA
CHB: Chronic hepatitis B
DC: Dendritic cell
ESCRT: Endosomal sorting complex required for transport
EV: Extracellular vehicles
HBV: Hepatitis B virus
HCV: Hepatitis C virus
HCC: Hepatocellular carcinoma
IFN: Interferon
miRNA: MicroRNA
MHC: Major histocompatibility complex,
MVB: Multivesicular body
NCR: Natural cytotoxicity receptors
NKT cell: Natural killer T cell
NK cell: Natural killer cell
NKG2D: Natural killer cell group 2D receptor
PRR: Pattern recognition receptor
ESCRT: Endosomal sorting complex required for transport
ORF: Open reading frames
SVR: Sustained viral response
TLRs: Toll-like receptors
TNF: Tumor necrosis factor
TRIF: Toll/IL-1 receptor domain-containing adaptor-inducing IFN-β
TSG101: Tumor susceptibility gene 101
VAPA: Vesicle-associated membrane protein-associated protein A
VPS: Vacuolar protein sorting-associated protein family
